# Drinking abstinence during a 3-month abstinence campaign in Thailand: weighted analysis of a national representative survey

**DOI:** 10.1186/s12889-019-8051-z

**Published:** 2019-12-16

**Authors:** Udomsak Saengow

**Affiliations:** Center of Excellence in Health System and Medical Research, Walailak University, 222 Thai Buri, Tha Sala, Nakhon Si Thammarat, 80160 Thailand

**Keywords:** Alcohol, Temporary abstinence, Campaign, Thailand

## Abstract

**Background:**

Temporary drinking abstinence campaigns have emerged globally in recent years. In Western countries, campaigns usually challenge drinkers to abstain for one month. In Thailand, the campaign called the Buddhist Lent Abstinence Campaign has been organized annually since 2003. The campaign encourages Thai people to abstain from drinking for three months during the Buddhist Lent period, which coincides with the monsoon season in Southeast Asia (around July–October). This study aimed to estimate the proportion and number of drinkers changing their drinking behaviours during the 3-month Thai abstinence campaign and to examine the determinants of abstinence.

**Methods:**

The 2016 Buddhist Lent Abstinence Evaluation Survey was analysed. The survey was a national representative survey of Thai populations aged ≥15 years. Weighted data were employed throughout the analysis. The number and proportion of drinkers changing their drinking behaviours were estimated. The determinants of alcohol abstinence during the campaign were explored using weighted logistic regression.

**Results:**

The prevalence of drinking in the Thai population was 34.3% (95% CI: 32.2–36.4%). A third of the current drinkers, equal to almost six million drinkers, abstained completely during the 3-month period. Another six million drinkers partially changed their drinking behaviours (16.3% abstained for a certain period, and 18.7% decreased the quantity of alcohol they consumed). The factors associated with abstinence included religion, occupation, drinking frequency prior to the campaign, type of beverages consumed, perceived harm from alcohol, exposure to campaign media, and making a public commitment.

**Conclusion:**

This study demonstrated the effectiveness of a temporary abstinence campaign in Thailand. The work is part of the growing global evidence on the effectiveness of this type of intervention. Temporary abstinence campaigns could be a potential approach to controlling alcohol consumption and related harms. Further research should focus on the long-term effects of such campaigns.

## Introduction

Recently, campaigns challenging individuals to temporarily refrain from drinking have emerged in many countries. Examples of those campaigns are “Dry January” in the UK, run by Alcohol Concern, with the first campaign in January 2013; “On The Dry” in Ireland, run by the Irish Heart Foundation, with the first event in January 2015; and “Dry July” in Australia, run by the Dry July Foundation, with the first public event in July 2008 [1–4]. All campaigns mentioned above called for one month of abstinence.

The temporary abstinence challenge might be considered a new type of social influence or mass media campaign. Conventional campaigns with methods such as resistance skills training and normative education attempt to alter attitudes towards drinking. The effects of such campaigns are controversial and have been regarded as ineffective strategies for reducing harm from drinking [5, 6]. The temporary abstinence challenge otherwise targets the actual behaviours of drinkers. It is designed to create a social contagion—the spread of an activity through a group of people—of drinking abstinence [7].

One study assessed the effects of Dry January in the UK. The study reported that campaign participants had decreased their drinking frequency, intoxication episodes, and drinking amount per drinking day in the sixth month after the campaign. It also observed an increase in drink refusal self-efficacy among campaign participants. The frequency of intoxication episodes prior to the campaign was the only predictor of successful completion of the one-month abstinence [7]. Hence, this type of campaign offers an alternative to conventional mass media campaigns.

A similar campaign has been implemented in Thailand since 2003, long before it emerged in Western countries. The campaign is called the Buddhist Lent Abstinence Campaign. The campaign organizers exploit the fact that the vast majority of Thais (93.6%) are Buddhists [8]. The campaign relates drinking abstinence to Buddhism’s concept of the Five Precepts (the basic code of ethics for lay people)—abstention from intoxication, including alcohol drinking, is one of the precepts [9]. Although most Thais describe themselves as Buddhist, the violation of the precept about drinking is not uncommon [10, 11]. To the author’s knowledge, no other countries with Buddhist majorities run this type of campaign. The campaign encourages Thai people to abstain from drinking for three months during the Buddhist Lent period, which concurs with the monsoon season in Southeast Asia (around July–October). This 3-month abstinence campaign is a part of wider social movement for health promotion in Thailand based on the concept of the mountain-moving triangle, i.e., three key actions—the creation of relevant knowledge, social movement, and political involvement—have to be taken to solve a difficult social problem or crisis [12–14]. The StopDrink Network Thailand, a not-for-profit organization working on alcohol-related problems, has run the campaign since its inception [15].

There are two main activities the campaign uses to promote alcohol abstinence. The first is a mass media campaign that advertises the campaign through TV broadcasting, radio, newspaper, billboard, and social media (including the campaign organizer’s website, online news websites, Facebook, and YouTube). The mass media campaign is carried out nationwide. The other activity is a community-based campaign. Local staff of the StopDrink Network Thailand who work with the community encourage community members to participate in the campaign. In some communities, there is a campaign kick-off ceremony that provides an opportunity for campaign participants to make a public commitment to the campaign, allowing other community members to monitor their drinking behaviour throughout the campaign period. Nevertheless, the campaign targets all drinkers and requires no formal registration. The campaign is mentioned under Strategy 2 (altering social norms towards alcohol and reducing drinking motivations) and Strategy 4 (promoting community-based solutions) of Thailand’s National Alcohol Strategy [16]. The campaign is funded primarily by the Thai Health Promotion Foundation.

To date, only two studies, mentioned earlier, have assessed the effectiveness of temporary abstinence campaigns, and both focused on the UK Dry January campaign. This study is the first to address temporary abstinence campaigns in Asian and middle-income countries, where drinking contexts and habits are different from those in high-income Western countries, using a national representative survey [5]. The objective of this study was to estimate the proportion and number of drinkers changing their drinking behaviours during the Thai 3-month abstinence campaign. Furthermore, the determinants of alcohol abstinence were examined.

## Methods

### Study design

This study used data from the 2016 Buddhist Lent Abstinence Evaluation Survey. The survey was conducted by the Research Centre for Social and Business Development and funded by the Center for Alcohol Studies for to the evaluation of the campaign. The survey used the multistage sampling method. First, the provinces were stratified into five strata: the Bangkok metropolitan region, central region, northern region, north-eastern region, and southern region. Three, two, two, three, and two provinces were selected from each stratum, respectively; a number of provinces from each stratum, and the probability of a province being selected within its strata was proportional to the population sizes of the strata and provinces. In the second and third stages, districts and sub-districts were randomly selected with a probability proportional to their size. In the fourth stage, subjects were randomly selected from among the sub-district civil registrations. Face-to-face interviews were conducted by trained interviewers during 1st–16th October 2016.

### Study subjects

Eligibility criteria for the survey subjects included an age ≥ 15 years and the ability to communicate in Thai. The sample size was computed to estimate the proportion of Thai people aged ≥15 who participated in the campaign using a formula for a finite population. The proportion was assumed to be 0.5 with a margin of error of 0.02. The 2015 population size of 52,618,286 was used in the sample size calculation [17]. A design effect of 1.4 and a level of significance of 0.05 were employed. The computed sample size was increased 25% to compensate for non-responses. The required sample size was 4202.

### Questionnaire

The survey questionnaire was divided into three parts. The first part included exposure to campaign media and opinion towards the campaign. The second part included drinking behaviours prior to and during the campaign. The third part included demographic data. Important questions about drinking behaviours include drinking status (drinking during the 12 months prior to the campaign and not drinking during the 12 months prior to the campaign), drinking frequency prior to the campaign (weekly, monthly, occasionally [less than once a month]), and beverage types drunk in the 12 months prior to the campaign (spirits, beer, wine, ready to drink [RTD], Thai frappe cocktail, and locally made alcohol). Thai frappe cocktail is a type of alcoholic beverage made by blending liquor with flavoured syrup, water, and ice. It is popular among teenagers and women [18].

Questions related to the campaign included changes in drinking behaviour, making a commitment to the campaign publicly, perceived impacts of abstinence, and exposure to campaign media. The drinking behavioural change variable comprised four levels: complete abstinence (abstained for three months), partial abstinence (abstained for a certain period), decreasing quantity (a reduced number of drinks per drinking occasion), and drinking as usual. Campaign kick-off ceremonies, which allowed members to make a commitment to the campaign publicly, were available only in some communities. Thus, the variable of making a public commitment included three levels: yes, no, and not available. Some who were exposed to the campaign media also forwarded the message to their family and peers. Survey subjects had three options when answering the question about exposure to campaign media: no exposure; yes, and forwarded the message to others; and yes, but did not forward the message to others. The questionnaire is included as a supplementary file (Additional file 1).

### Data analysis

Weighted data were employed throughout the analysis. Data were weighted according to the sampling scheme to represent the Thai population aged ≥15. Those who did not drink during the 12 months prior to the campaign were excluded from the analysis, as they could not be counted as campaign participants. A composite variable indicating socio-economic status (SES)—namely, the SES index—was created by adding scores from two variables: education (grade 6 or lower = 0, grade 7–12 = 1, and college or higher = 2) and monthly income (5000 Thai baht [THB] or less = 0, 5001–10,000 THB = 1, 10,001 THB or more = 2). The SES index therefore had five levels, with 0 indicating the lowest SES level and 4 indicating the highest SES level. The SES index was used as an independent variable instead of the education or income variables in the regression analysis.

The drinking behaviour change variable was further dichotomised and used as a dependent variable of the regression. A participant with complete abstinence or partial abstinence was classified as an “abstainer”. A participant with a decreasing quantity or drinking as usual was classified as a “drinker”. The decreasing quantity group was grouped with the drinking as usual group to avoid social desirability bias [19]. The determinants of alcohol abstinence during the campaign were explored using weighted logistic regression. The interaction between the making a public commitment variable and drinking frequency prior to the campaign was tested by adding the interaction term to the logistic model. This was done to examine whether making a public commitment could modify the relationship between the previous drinking behaviour and behaviour change variables—an association found in a previous study [7].

## Results

The total number of survey subjects was 4296. Among those, 1486 had drunk in the 12 months prior to the campaign (current drinkers). Using weighted data, 17,922,215 Thai drinkers were used, and the estimated drinking prevalence of the Thai population was 34.3% (95% CI: 32.2–36.4%). Only current drinkers were included in the further analysis.

Table 1 shows the characteristics of the respondents who were drinkers. Most Thai drinkers were men in working age groups and were concentrated in the higher half of the SES scale. Nearly all Thai drinkers were Buddhists. In the 12 months prior to the campaign, a third of the drinkers drank at least once every week, and a third drank less than once a month. Beer was the most popular alcoholic beverage, followed by spirits and locally made alcohol. More than 80% of the drinkers perceived that alcohol is very harmful. A minority of drinkers (15.5%) were not exposed to the campaign media. Less than one-fifth had made a public commitment to the campaign.

**Table 1:** Characteristics of survey respondents who were drinkers (weighted)

Characteristic	%	95% CI
Gender		
Man	69.8	66.6–73.1
Woman	30.2	26.9–33.4
Age (years)		
≤ 20	10.9	8.7–13.1
21–30	19.8	17.2–22.4
31–45	37.1	33.3–40.8
46–60	27.4	24.0–30.9
> 60	4.8	3.6–6.1
Religion		
Buddhism	99.4	99.0–99.7
Islam	0.2	0.0–0.4
Others	0.4	0.1–0.7
SES index		
0 (lowest SES)	6.8	5.1–8.5
1	18.0	14.9–21.2
2	23.7	20.7–26.7
3	29.7	26.0–33.3
4 (highest SES)	21.8	18.9–24.7
Occupation		
Public employee	8.4	5.9–11.0
Business owner	29.6	25.9–33.3
Private employee	15.1	12.6–17.5
Worker in the informal sector	31.3	28.1–34.6
Unemployed/retired	5.0	3.6–6.3
Student	10.6	8.4–12.9
Drinking frequency prior to the campaign		
Weekly	33.5	29.9–37.0
Monthly	31.2	27.7–34.8
Occasionally	35.3	31.7–38.9
Type of beverages consumed prior to the campaign		
Spirits	60.2	56.5–63.9
Beer	76.6	73.2–80.1
Wine	7.2	5.5–8.9
Ready to drink (RTD)	5.2	3.7–6.6
Thai frappe cocktail	6.3	4.4–8.2
Locally made alcohol	14.1	11.6–16.5
Perceived harm from alcohol		
Very harmful	83.3	80.7–85.9
Little or no harm	16.7	14.1–19.3
Perceived impacts of abstinence		
Save money	80.7	77.0–84.5
Improve physical health	79.6	75.5–83.7
Improve mental health	46.6	41.9–51.2
Decrease problems in family	30.3	26.0–34.5
Exposure to campaign media		
No exposure	15.5	13.1–17.8
Yes, and forwarding the message to others	68.7	65.2–72.2
Yes, but not forwarding the message to others	15.8	12.8–18.9
Making a public commitment		
Yes	17.1	13.9–20.3
No	47.2	43.4–50.9
Not available	35.8	32.2–39.3

As shown in Table 2, a third of the current drinkers (32.2%), which is equal to almost six million drinkers, abstained completely during the 3-month period. The other six million drinkers partially changed their drinking behaviour: 16.3% abstained for a certain period, and 18.7% decreased the quantity of alcohol they consumed. A third of the drinkers continued their drinking habits.

**Table 2:** Estimated prevalence and number of drinkers with certain drinking behaviours during the campaign

Drinking behaviour	Prevalence (95% CI)	Estimated number (persons)
Current drinkers who abstained completely during the 3-month period	32.2% (28.7–35.7%)	5,772,905
Current drinkers who partially abstained	16.3% (13.7–19.0%)	2,923,663
Current drinkers who reduced their number of drinks per drinking occasion	18.7% (15.4–21.9%)	3,345,251
Current drinkers who continued drinking as usual	32.8% (29.4–36.2%)	5,880,396

Table 3 demonstrates the rates of abstinence by each characteristic. Subgroups with a significantly high rate of abstinence during the campaign included occasional drinkers and those making a public commitment to the campaign. Subgroups with abstainers being less than 30% included those having religions other than Buddhism, frequent drinkers, and those who had drunk a Thai frappe cocktail in the 12 months prior to the campaign.

**Table 3:** Rate of abstinence during the campaign by demographic characteristics and drinking behaviour

Characteristic	% abstinence	P-value
Gender		0.016*
Man	45.7	
Woman	55.1	
Age (years)		0.341
≤ 20	51.4	
21–30	45.7	
31–45	45.8	
46–60	51.0	
> 60	60.5	
Religion		< 0.001*
Buddhism	48.7	
Islam	11.9	
Other	17.4	
SES index		0.007*
0 (lowest SES)	59.3	
1	51.9	
2	48.3	
3	44.0	
4 (highest SES)	47.7	
Occupation		0.096
Public employee	44.6	
Business owner	44.1	
Private employee	43.1	
Worker in the informal sector	54.9	
Unemployed/retired	60.8	
Student	47.4	
Drinking frequency prior to the campaign		< 0.001*
Weekly	27.1	
Monthly	54.1	
Occasionally	63.3	
Type of beverages consumed prior to the campaign		
Spirits	40.4	< 0.001*
Beer	50.0	0.186
Wine	37.3	0.047*
Ready to drink (RTD)	53.5	0.485
Thai frappe cocktail	28.7	0.004*
Locally made alcohol	32.4	< 0.001*
Perceived harm from alcohol		< 0.001*
Very harmful	51.7	
Little or no harm	32.6	
Exposure to campaign media		< 0.001*
No exposure	37.8	
Yes, and forwarding the message to others	55.2	
Yes, but not forwarding the message to others	31.1	
Making a public commitment		< 0.001*
Yes	68.3	
No	43.7	
Not available	45.6	

*Note.* *: P-value < 0.05

Table 4 shows the results from the weighted logistic regression. After adjustment, the factors associated with abstinence included religion, occupation, drinking frequency prior to the campaign, type of beverages consumed, perceived harm from alcohol, exposure to campaign media, and making a public commitment. Those with religions other than Buddhism tended not to abstain. Informal sector workers were more than two times more likely to abstain when compared to public employees. Those who drank less frequently had a considerably higher likelihood of abstaining during the campaign when compared to those who drank every week. Drinking spirits or Thai frappe cocktail halved the chance of abstinence. Those who perceived alcohol to be less harmful were less likely to abstain. Forwarding the campaign message to others and making a public commitment increased the likelihood of abstinence. The interaction between the making a public commitment variable and drinking frequency prior to the campaign in terms of campaign participation was not statistically significant (*p*-value = 0.293; results not shown).

**Table 4:** Factors associated with abstinence during the campaign

Variable	adj. OR (95% CI)	P-value (LR test)
Gender [reference: woman]		0.887
Man	1.03 (0.69, 1.52)	
Age [reference: ≤ 20]		0.696
21–30	0.79 (0.41, 1.54)	
31–45	0.77 (0.38, 1.56)	
46–60	0.72 (0.35, 1.5)	
> 60	1.17 (0.48, 2.91)	
Religion [reference: Buddhism]		0.040*
Islam	0.21 (0.04, 1.11)	
Other	0.26 (0.04, 1.87)	
SES index [reference: 0 (lowest SES)]		0.103
1	0.83 (0.32, 2.13)	
2	0.71 (0.27, 1.84)	
3	0.73 (0.28, 1.95)	
4 (highest SES)	0.89 (0.32, 2.49)	
Occupation [reference: Public employee]		0.038*
Business owner	1.35 (0.64, 2.89)	
Private employee	1.27 (0.60, 2.69)	
Worker in the informal sector	2.47 (1.10, 5.54)*	
Unemployed/retired	2.36 (0.85, 6.59)	
Student	1.52 (0.55, 4.17)	
Marital status [reference: Married]		0.465
Other	1.16 (0.48, 2.79)	
Single	0.95 (0.63, 1.45)	
Residence [reference: Rural area]		0.265
Urban area (outside Bangkok)	1.31 (0.90, 1.89)	
Bangkok	1.14 (0.71, 1.85)	
Drinking frequency prior to the campaign [reference: Weekly]		< 0.001*
Monthly	2.85 (1.93, 4.19)*	
Occasionally	4.12 (2.70, 6.29)*	
Type of beverages consumed prior to the campaign period		
Spirits [reference: No]		
Yes	0.56 (0.40, 0.78)*	0.001*
Beer [reference: No]		
Yes	1.10 (0.73, 1.66)	0.644
Wine [reference: No]		
Yes	0.50 (0.22, 1.15)	0.102
Ready to drink (RTD) [reference: No]		
Yes	1.59 (0.87, 2.91)	0.131
Thai frappe cocktail [reference: No]		
Yes	0.42 (0.18, 0.98)*	0.042*
Locally made alcohol [reference: No]		
Yes	0.76 (0.47, 1.23)	0.267
Perceived harm from alcohol [reference: Very harmful]		0.002*
Little or no harm	0.48 (0.29, 0.78)*	
Exposure to campaign media [reference: No]		0.001*
Yes, and forwarding the message to others	1.68 (1.12, 2.54)*	
Yes, but not forwarding the message to others	0.89 (0.51, 1.55)	
Making a public commitment [reference: No]		< 0.001*
Yes	2.15 (1.34, 3.47)*	
Not available	1.03 (0.72,1.48)	

*Note.* *: P-value < 0.05

## Discussion

This study demonstrates that the 3-month abstinence campaign in Thailand led to the complete abstinence of approximately six million drinkers (one-third of the current drinkers) and the partial abstinence of three million other drinkers. Drinking behaviours prior to the campaign (frequency and types of consumed beverages), campaign activities (making a public commitment and campaign media exposure), perception about harm from drinking, and demographic characteristics were associated with drinking behaviours during the campaign period.

The 3-month abstinence campaign in Thailand achieved a higher rate of participation (via participants stating that they had abstained or reduced their consumption) compared to the UK “Dry January” campaign (1-month abstinence challenge): the participation rate was 67.2% in Thailand and 11% in the UK 2016 campaign [20]. Thailand is a relatively dry country; only a third of the population aged 15 and above are current drinkers. In the UK, the prevalence of current drinkers was 83.9% in 2010 [21]. Drinking prevalence could reflect the norms in different countries regarding drinking [22]. In Thailand, where drinking is not the norm, encouraging people to abstain from drinking might be easier. The extensive community-based activities of the Thai campaign might also contribute to the high rate of participation.

One distinct feature of the Thai campaign is that it incorporates the Buddhism concept of the Five Precepts and Buddhist Lent into the campaign. This religious aspect is designed to enhance participation and compliance with the campaign by taking advantage of the fact that most Thais are Buddhists [14]. The findings of this study indicated one weakness of this approach: people of other religions were far less likely to pay attention to the campaign. Furthermore, there might also be an issue regarding the generalizability of this approach. Nevertheless, previous studies have demonstrated the effectiveness of religion-based or faith-based approaches in campaigns to promote healthy behaviours, i.e., HIV/AIDS prevention and weight loss; such campaigns have adopted ideas from Christianity [23, 24]. Thus, the effectiveness of such an approach is not specific to Buddhism. The general lesson drawn from this finding is that a campaign of this kind can be modified to suit local contexts, cultures, or faiths.

Making a public commitment to the campaign boosted the chance of abstinence. Making a commitment either privately or publicly has been shown to be an effective strategy for short- and long-term changes in behaviours regarding household conservation (recycle, energy saving, and water saving) [25]. The explanation is that the act of making such a commitment changes a person’s internal motivation (i.e., self-concept and attitude) in line with the committed behaviour [26]. For those making a public commitment, there is an additional effect from social pressure [27]. Both UK Dry January and Dry July in Australia ask participants to enrol in the campaign using a mobile application [1, 2]. The enrolment can be thought of as a commitment-making process. Campaign participants evidently shared their campaign activities on social network platforms [20]. This resembles the public commitment in the Thai campaign. Making a public commitment in the Thai campaign can only be done at campaign kick-off ceremonies, which are available only in some communities. The adoption of an online application for campaign enrolment with an option to share the commitment on social media platforms could enhance the complete abstinence rate and encourage more people to participate.

An association between drinking behaviours prior to the campaign period and successful abstinence was reported in a previous study from the UK [7]. Those drinking more frequently were less likely to abstain, which could be due to the dependence effects of alcohol. Immediate abstinence from drinking can trigger withdrawal symptoms in alcohol-dependent individuals [5]. For the alcohol-dependent group, encouraging them to access treatment is more appropriate than complete abstinence. The relationship between the types of beverages consumed and abstinence might reflect wider contexts of drinking. Each beverage was consumed more commonly in certain contexts and led to different consequences [28]. Beverage types were associated with traits of drinkers as well [29]. In Thailand, spirits and Thai frappe cocktails might be consumed in a more regular and/or social manner or preferred by those who are relatively difficult to persuade; there were lower rates of abstinence in drinkers of both of these beverages.

The primary strength of this study is the usage of data from a national representative survey. This study is also the first systematic attempt to assess and report a temporary abstinence campaign from an Asian and middle-income country. This study has some limitations. The assessment of drinking behaviours was based on self-report and was likely suffer from social desirability effects, given the involvement of religion. To mitigate this effect, those reducing their consumption (but not abstaining) were grouped with those who were drinking as usual. The survey contained no follow-up data to assess whether the abstainers consumed more than usual after the campaign ended (rebound effect). Regarding this point, findings from the UK study indicated that such a rebound effect affected only a small portion of campaign participants [7].

## Conclusions

In conclusion, this study, together with findings from previous UK studies [7, 30], sheds light on the effectiveness and generalizability of a temporary abstinence campaign. The temporary abstinence campaign could be a novel approach to contributing to achieving the WHO NCD target for alcohol (Target 2: At least a 10% relative reduction in the harmful use of alcohol) in addition to good-buy and best-buy measures [31, 32]. Knowledge about the determinants of abstinence could be used to improve the campaign administration, as discussed above. Further research should focus on the long-term effects of the campaign, comparing alcohol-related problems among participants and non-participants of the campaign, and how campaign participants cope with changes in their routine in relation to drinking.

## Supplementary information

**Additional file 1.** Survey questionnaire. English translation of the questionnaire used in the survey (originally in Thai)

## Data Availability

The data that support the findings of this study are available from the Center for Alcohol Studies, Thailand, but restrictions apply to the availability of these data, which were used under license for the current study, and are therefore not publicly available. Data are, however, available from the authors upon reasonable request and with permission of the Center for Alcohol Studies, Thailand.

## Abbreviations

RTD: Ready to drink
SES: Socio-economic status
THB: Thai baht
