# Supplementary material for: Drinking abstinence during a 3-month abstinence campaign in Thailand: weighted analysis of a national representative survey
Source: BMC Public Health. 2019 Dec 16;19:1688. doi: 10.1186/s12889-019-8051-z (PMC6916250; doi:10.1186/s12889-019-8051-z)
Supplement: Supplementary file 1 — Additional file 1. Survey questionnaire. English translation of the questionnaire used in the survey (originally in Thai) [file 12889_2019_8051_MOESM1_ESM.pdf]

ID [ ] [ ] [ ] [ ] [ ]

Questionnaire: "Buddhist Lent Abstinence Evaluation Survey 2016"

	**Section 1**
1.	In 2016 prior to the Buddhist Lent, have you seen/been exposed to Buddhist Lent Alcohol Abstinence campaign's advertising?<br><br>○ 1. No (skip to question 3)<br><br>○ 2. Yes and also forwarded campaign's advertising or materials to others<br><br>○ 3. Yes, but did not forward campaign's advertising or materials to others
2.	If you answer 'Yes' in Question 1, please select media which you have been exposed to the campaign advertising (choose all that apply)?<br><br>☐ 1. Television ☐ 2. Large billboard<br><br>☐ 3. Small billboard/placard ☐ 4. Radio<br><br>☐ 5. Website/social media ☐ 6. Newspaper/magazine<br><br>☐ 7. Brochure ☐ 8. Family/friend/acquaintance<br><br>☐ 9. Local event/campaign-related activity
3.	Do you agree that asking people to abstain from drinking for three months during the Buddhist Lent period can reduce alcohol consumption?<br><br>○ 1. Agree<br><br>○ 2. Disagree
	**Section 2**
4.	During past 12 months before the start of this year Buddhist Lent, did you drink alcoholic beverage?<br><br>○ 1. No<br><br>○ 2. Yes
5.	How frequent did you drink during the period in question 4?<br><br>○ 1. Less than monthly ○ 2. Monthly (1-3 days/ month)<br><br>○ 3. Weekly (1-4 days/week) ○ 4. Daily (5-7 days/week)
6.	Type of alcoholic beverages consumed (choose all that apply):

	☐ 1. Spirit ☐ 2. Beer<br>☐ 3. Wine ☐ 4. Ready-to-drink (RTD)<br>☐ 5. Thai frappe cocktail ☐ 6. Locally-made alcohol
7.	How much you typically paid per drinking occasion (including food, tip, and other expenses)?<br>……………………………… Thai baht
8.	Have you ever thought of quitting alcohol permanently?<br>○ 1. No ○ 2. Yes
9.	What was your drinking behavior during this year Buddhist Lent period (20th July–16th October)?<br>○ 1. Abstain from drinking throughout the Lent period<br>○ 2. Abstain from drinking for a certain period during the Lent<br>○ 3. Reduced a number of drinks per drinking occasion<br>○ 4. Drink as usual
10.	What was impact of the abstinence that happened to you (choose all that apply)?<br>☐ 1. Saved money<br>☐ 2. Improved physical health<br>☐ 3. Improved mental health<br>☐ 4. Decreased problems in family
11.	Do you intend to abstain from drinking during the Lent period next year?<br>○ 1. Yes, I will abstain throughout the Lent period.<br>○ 2. Yes, I will abstain for a certain period during the Lent.<br>○ 3. I will not abstain, but reduce a number of drinks per drinking occasion<br>○ 4. No, I will drink as usual.
12.	Have you made public commitment to abstain for drinking during the Lent period?<br>○ 1. Yes<br>○ 2. No

	○ 3. Not available (no campaign activity in my residential area)
13.	In your opinion, how much can alcohol pose harm to your health?<br>○ 1. Alcohol is very harmful to health.<br>○ 2. Alcohol poses little harm to health.<br>○ 3. Alcohol poses no harm to health.
	**Section 3**
14.	Gender<br>○ 1. Male<br>○ 2. Female
15.	Age<br>…………… years
16.	Level of education<br>○ 1. Grade 6 or lower<br>○ 2. Grade 7-12<br>○ 3. College and higher
17.	Religion<br>○ 1. Buddhism<br>○ 2. Christianity<br>○ 3. Islam<br>○ 4. Others
18.	Occupation<br>○ 1. Public employee<br>○ 2. Business owner<br>○ 3. Private employee<br>○ 4. Worker in informal sector

	○ 5. Unemployed/retired<br><br>○ 6. Student
19	Average monthly income<br><br>…………………… Thai baht
20	Residence:<br><br>District ………………………………………<br><br>Province …………………………………….
